# The impact of metformin use on survival in prostate cancer: a systematic review and meta-analysis

**DOI:** 10.18632/oncotarget.22117

**Published:** 2017-10-31

**Authors:** Yao Xiao, Lei Zheng, Zubing Mei, Changbao Xu, Changwei Liu, Xiaohan Chu, Bin Hao

**Affiliations:** ^1^ Department of Urology, The Second Affiliated Hospital of Zhengzhou University, Zhengzhou, Henan Province, China; ^2^ Department of Endocrinology, The First Affiliated Hospital of Chinese PLA General Hospital, Beijing, China; ^3^ Department of Anorectal Surgery, Shuguang Hospital, Shanghai University of Traditional Chinese Medicine, Shanghai, China

**Keywords:** prostate cancer, prognosis, metformin, meta-analysis, survival

## Abstract

**Background:**

Metformin has been implicated to reduce the risk of prostate cancer (PCa) beyond its glucose-lowering effect. However, the influence of metformin on prognosis of PCa is often controversial.

**Results:**

A total of 13 cohort studies encompassing 177,490 individuals were included in the meta-analysis. Data on overall survival (OS) and cancer-specific survival (CSS) was extracted from 8 and six studies, respectively. Comparing metformin users with non-metformin users, the pooled hazard ratios (HRs) for OS and CSS were 0.79 (95% confidence interval [CI] 0.63–0.98) and 0.76 (95% CI 0.57–1.02), respectively. Subgroup analyses stratified by baseline charcteristics indicated significant CSS benefits were noted in studies conducted in USA/Canada with prospective, large sample size, multiple-centered study design. Five studies reported the PCa prognosis for recurrence-free survival (RFS) and metformin use was significantly associated with patient RFS (HR 0.74, 95% CI, 0.58–0.95).

**Methods:**

Relevant studies were searched and identified using PubMed, Embase and Cochrane databases from inception through January 2017, which investigated associations between the use of metformin and PCa prognosis. Combined HRs with 95% CI were pooled using a random-effects model. The primary outcomes of interest were OS and CSS.

**Conclusions:**

Our findings provide indication that metformin therapy has a trend to improve survival for patients with PCa. Further prospective, multi-centered, large sample size cohort studies are warranted to determine the true relationship.

## INTRODUCTION

Biguanides, commonly known as metformin, are one type of the most widely prescribed drugs mainly to lower blood glucose for patients with type 2 diabetes. Experimental studies have shown that metformin has anti-neoplastic effects in several malignant tumors, including breast cancer, pancreatic cancer, and prostate cancer (PCa) [1–3].

Metformin has been implicated to restrain mitochondrial complex [1], reducing mitochondrial ATP production, leading to cellular energetic stress [3], which can activate AMPK, resulting in the inhibition of tumor growth through an anti-proliferative phenotype [3, 4]. Metformin can also act as a chemosensitizer. In breast cancer xenograft models, metformin has been shown to enhance the effect of chemotherpy and prolong remission in breast cance cell line. In colon cancer cell lines, metformin can enhance the chemosensitivity of 5-fluorouracil and oxaliplatin [5, 6]. Moreover, metformin has also been shown to improve survival in diabetic patients with advanced endometrial cancer and non-small cell lung cancer [7, 8].

The effect of metformin use in PCa has been examined by many studies [9–22]. Although it has been found in some studies that metformin showed no significant positive association with PCa outcomes [10, 15–17, 22], while still others reported negative [11, 12, 14, 18–21]. Several studies have especially reported that metformin is associated with reduced risk and mortality of PCa [9, 11, 12, 18, 19, 21, 23].

However, these results were controversial. Therefore, we updated the systematic review and meta-analysis to reappraise the prognostic value of metformin in PCa.

## RESULTS

### Description of the search and selection of studies

**Figure 1:**
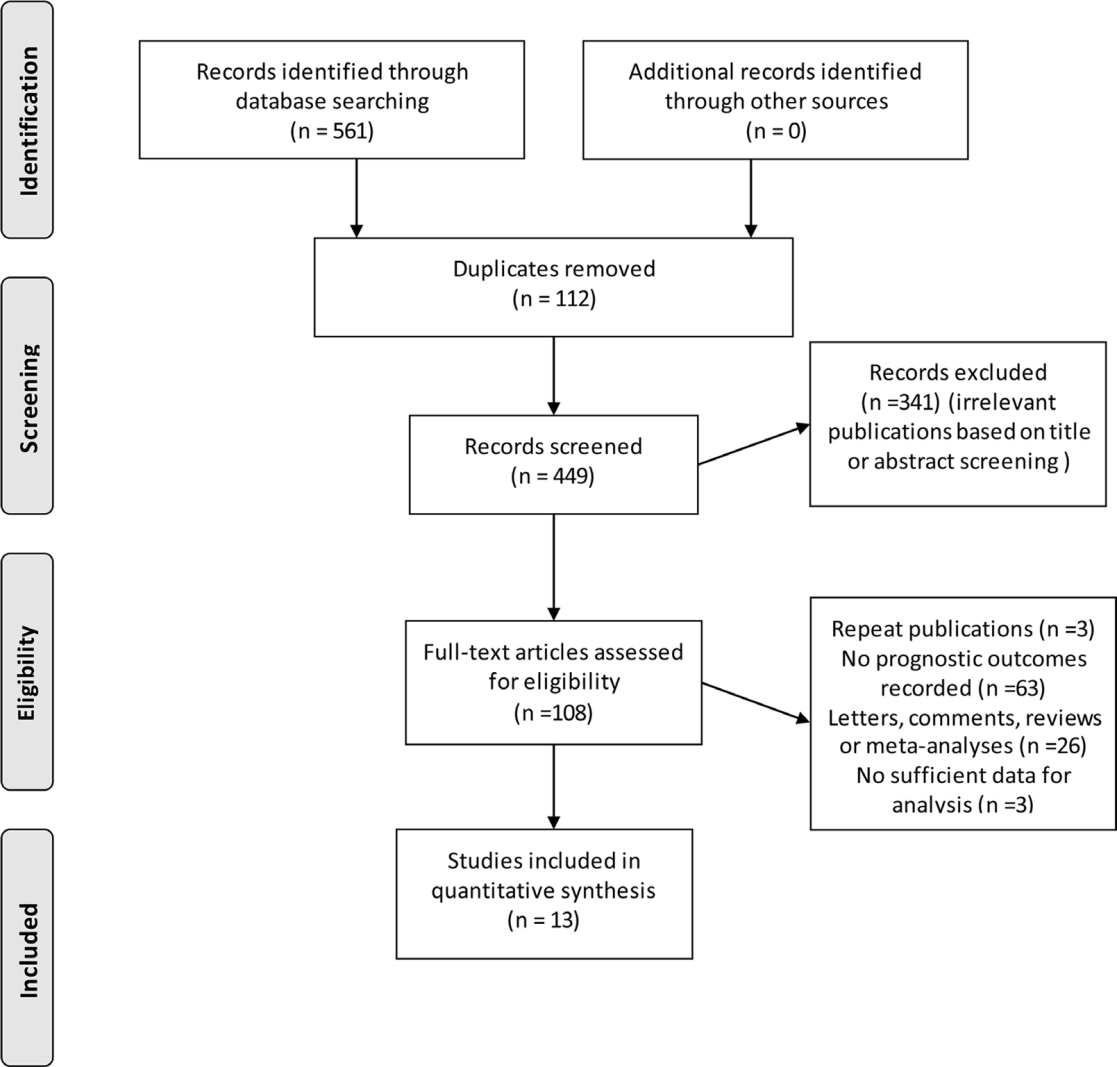
Flow diagram of study selection process investigating effect of metformin use on prostate cancer prognosis

A total of 561 citations were identified for eligibility through the systematic literature search. After exclusion of duplicate publications and full text review of the relevant studies, A total of 13 cohort studies encompassing 177,490 individuals, with a mean sample size of 13,653 (range 250 to 105,245) were included in the quantitative synthesis. Data on overall survival (OS) and cancer-specific survival (CSS) were available from 8 and 6 studies, respectively [10–22] (Figure 1 and Supplementary Tables 1–4).

### Study characteristics

Table 1 provides the baseline characteristics of each study that met our inclusion criteria. All studies were published between 2010 and 2016 in English peer-reviewed journals. Five of the included studies were population-based cohort studies and eight were hospital-based cohort studies. Nine studies has retrospective designs, and four studies has prospective designs. Ten studies were performed in USA or Canada, two in Europe and one in Asia. Five studies involved single-center data, whereas eight were multi-center studies. Assessment of methodological quality by NOS yielded a mean score of 7 (range, 6 to 9), and 8 of 10 studies had a score of 7 or above (Table 2).

**Table 1:** Baseline characteristics of included studies investigating the survival outcomes of metformin use for PCa patients

First author	Country	Inclusion period	Source of data	Study design	Study setting	No. of hospitals involved	Sample size	Metformin user/non-user	Median follow-up	Survival endpoints	Study quality
(year)									(years)		
Mayer	Canada	2005–2012	Several Ontario administrative health care databases	Retrospective	Population-based	Multiple centers	2,832	359/1,247	NR	CSS,OS	7
2016											
Chong	USA	NR	Tumor Registry at the Memphis Veterans Affairs Medical Center	Retrospective	Hospital-based	Single center	287	138/149	NR	OS,RFS	7
2016											
Reznicek	USA	2002–2010	Baltimore Veterans Administration	Retrospective	Hospital-based	Single center	1,155	NR	5.5(Me)	OS	8
2015											
Randazzo	Switzerland	1998–2003	ERSPC Aarau	Prospective	Population-based	Multiple centers	10,311	150/4164	7.6(Me)	OS,CFS	8
2015											
Lu-Yao	USA	2007–2009	Surveillance, Epidemiology, and End Results-Medicare linked data	Retrospective	Population-based	Multiple centers	22,110	NR	NR	CSS	7
2015											
Lee	Korea	2006–2013	Committee on the Ethics of the Seoul National University Bundang Hospital	Retrospective	Hospital-based	Single center	746	135/74	NR	RFS	8
2015											
Kaushik	USA	1997–2010	Mayo Clinic electronic medical record	Retrospective	Hospital-based	Single center	12,052	562/323	5.1(Me)	RFS,CFS, OS	9
2014											
Bensimon	UK	1998–2009	UK NCDR, the CPRD, the HES database, and the Office for National Statistics database	Retrospective	Population-based	Multiple centers	15,940	242/138	3.7(M)	CSS,OS	7
2014											
Spratt	USA	1992–2008	Memorial Sloan-Kettering Cancer Center	Retrospective	Hospital-based	Single center	3,045	157/162	8.7(Me)	CSS	8
2013											
Margel	Canada	1997–2008	Several database*	Retrospective	Population-based	Multiple centers	105,245	1619/2218	4.64(Me)	CSS,OS	8
2013											
Spratt	USA	1993–2009	NR	Retrospective	Hospital-based	Single center	2,901	157/159	13.4(Me)	CSS	6
2012											
He	USA	1999–2008	Data from University of Texas M. D. Anderson Cancer Center	Retrospective	Hospital-based	Single center	250	NR	NR	OS	6
2011											
Patel	USA	1990–2009	Columbia University Urologic Oncology Database	Retrospective	Hospital-based	Single center	616	112/98	NR	RFS	6
2010											

Abbreviations: BCR = biochemical recurrence; BMI = body mass index; CFS = cancer-free survival; CPRD = Clinical Practice Research Datalink; CIHI = Canadian Institute for Health Information; CSS = cancer specific survival; ERSPC = European Randomized Study of Screening for Prostate Cancer; HES = Hospital Episode Statistics; M = mean; Me = median; NCDR = National Cancer Data Repository; NR = not report; OS = overall survival; PCa = prostate cancer; RFS = recurrence-free survival.

*the Ontario Cancer Registry, the Ontario Diabetes Database, the Ontario Health Insurance Plan, the CIHI Discharge Abstract Database, the CIHI National Ambulatory Care Reporting System, the Registered Persons Data Base, the Ontario Drug Benefit database.

**Table 2:** Methodological quality of included studies based on the Newcastle–Ottawa Scale for cohort studies

Study	Design	Selection	Comparability	Outcome/exposure	Overall quality (max 9)
Mayer (2016)	Cohort	***	**	**	7
Chong (2016)	Cohort	****	**	*	7
Reznicek (2015)	Cohort	****	**	**	8
Randazzo (2015)	Cohort	***	**	***	8
Lu-Yao (2015)	Cohort	****	**	*	7
Lee (2015)	Cohort	****	**	***	9
Kaushik (2014)	Cohort	****	**	***	9
Bensimon (2014)	Cohort	***	**	**	7
Spratt (2013)	Cohort	****	**	**	8
Margel (2013)	Cohort	***	**	***	8
Spratt (2012)	Cohort	***	**	*	6
He (2011)	Cohort	***	**	*	6
Patel (2010)	Cohort	***	**	*	6

*Study quality assessment of observational studies performed using the Newcastle–Ottawa scale (each asterisk represents if individual criterion within the subsection were fulfilled).

### Metformin use and PCa survival

#### Metformin use and patient overall survival

**Figure 2:**
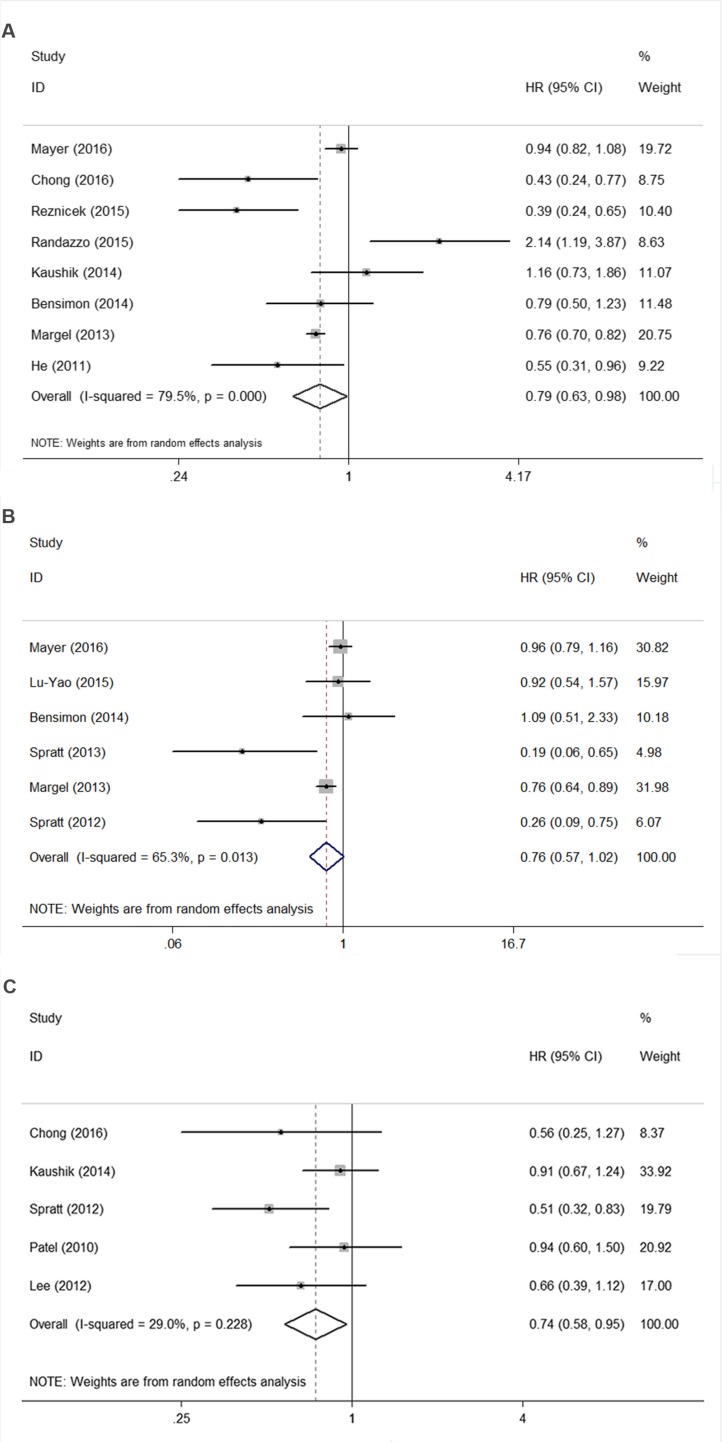
Funnel plot of studies investigating association between metformin use and (**A**) overall survival, (**B**) cancer-specific survival, (**C**) recurrence-free survival.

As shown in Figure 2A, the pooled hazard ratio (HR) for the OS comparing metformin use versus non-use was 0.79 (95% CI 0.63–0.98), and there was significant inter-study heterogeneity (*I*^2^ = 79.5%, *P* < 0.001). The subgroup analysis limited study region to USA/Canada showed similar result (*n* = 6, HR 0.72, 95% CI 0.57–0.90). We also found that studies with retrospective design, sample size less than 10,000, hospital-based study, single center study, with patients including only diabetics and metformin use calculated as ever versus never use have similar results with the main analysis. However, due to the limited studies included in some subgroups, though the trend of the survival benefits were noted, significant differences were not reached (Table 3A).

**Table 3A:** Subgroup analysis of overall survival

	HR	95% CI	Degree of heterogeneity (*I*^2^ statistics; %)	*P*-value	No. of included Studies
Study quality					
Score≥8	0.9	0.54 to 1.50	86.2	<0.001	4
Score<8	0.7	0.49 to 1.00	68.8	0.022	4
Study region					
USA/Canada	0.72	0.57 to 0.90	78.4	<0.001	6
Europe	1.28	0.48 to 3.38	85.6	0.008	2
Study design					
Prospective	2.14	1.19 to 3.87	–	–	1
Retrospective	0.73	0.59 to 0.89	74.1	0.001	7
Sample size					
<10000	0.56	0.33 to 0.95	84.2	<0.001	4
≥10000	1.04	0.69 to 1.55	79.2	0.002	4
Study setting					
Hospital-based	0.58	0.34 to 0.97	74.7	0.008	4
Population-based	0.93	0.73 to 1.19	82.8	0.001	4
Number of hospital					
Single	0.58	0.34 to 0.97	74.7	0.008	4
Multiple	0.93	0.73 to 1.19	82.8	0.001	4
Diabetics only					
Yes	0.66	0.50 to 0.87	66.2	0.011	6
No	1.35	0.61 to 3.00	85.9	0.008	2
Effect estimates					
Time varing HR	0.84	0.65 to 1.08	83.2	<0.001	6
Not HR	0.6	0.33 to 1.09	61.8	0.105	2
Metformin use					
Cumulative use	0.78	0.51 to 1.19	83.2	<0.001	6
Ever vs never use	0.76	0.70 to 0.82	0	0.868	2
Statistical method					
Time varying cox regression	0.82	0.54 to 1.25	82.7	<0.001	5
Single regression	0.74	0.49 to 1.11	70.8	0.032	3

### Metformin use and patient cancer-specific survival

Figure 2B showed that the pooled HR for the CSS comparing metformin use versus non-use was 0.76 (95% CI 0.57–1.02), and there was significant inter-study heterogeneity (*I*^2^ = 65.3%, *P* = 0.013). The subgroup analysis limited study region to USA/Canada showed similar result with boundary survival benefit (*n* = 5, HR 0.73, 95% CI 0.53–1.00). We also find that studies with prospective design, larger sample size more than 10,000, population-based study and multiple center study have similar trends of survival benefits for metformin use with the main analysis. Due to the limited studies included in the main analysis and some subgroups, through the trend of the survival benefits were found, further large prospective studies need to be conducted to test this association (Table 3B).

Five studies investigated the association between metformin use and recurrence-free survival (RFS), we found that metformin use was significant associated with improved RFS for PCa Patients (*n* = 5, HR 0.74, 95% CI 0.58–0.95).

**Table 3B:** Subgroup analysis of cancer-special survival

	HR	95% CI	Degree of heterogeneity (*I*^2^ statistics; %)	*P*-value	No. of included Studies
Study quality					
Score ≥ 8	0.43	0.11 to 1.64	80.4	0.024	2
Score < 8	0.85	0.58 to 1.24	48.5	0.12	4
Study region					
USA/Canada	0.73	0.53 to 1.00	71.2	0.008	5
Europe	1.09	0.51 to 2.33		<0.001	1
Study design					
Prospective	–	–	–	–	0
Retrospective	0.76	0.57 to 1.02	65.3	0.013	6
Sample size					
<10000	0.4	0.13 to 1.3	83.6	0.002	3
≥10000	0.78	0.67 to 0.91	0	0.548	3
Study setting					
Hospital-based	0.23	0.10 to 0.50	0	0.7	2
Population-based	0.86	0.74 to 1.00	21.2	0.283	4
Number of hospital					
Single	0.86	0.74 to 1.00	21.2	0.283	4
Multiple	0.23	0.10 to 0.50	0	0.7	2

### Sensitivity analyses and publication bias

The tests for funnel plot asymmetry in OS and CSS subset indicated the absence of publication bias, which were further confirmed by Egger’s test (*P* = 0.69 for OS, *P* = 0.32 for CSS), and Begg’s test (*P* = 1.00 for OS, *P* = 0.26 for CSS). The adjusted estimates calculated using the trim-and-fill method were similar with the original analyses for both OS and CSS (Supplementary Table 5). We did not explore the publication bias for RFS due to the limited number of studies involved.

## DISCUSSION

### Principal findings of this study

This present systematic review and meta-analysis represents the most comprehensive review to date on the association between metformin use and PCa prognosis by including 13 cohort studies enrolling 177,490 individuals. Overall, we find that metformin intake has a trend to improve survival for patients with PCa in terms of OS, CSS and RFS. Significant CSS benefits were noted in studies conducted in USA/Canada with prospective, large sample size, multiple-centered study design.

### Comparisons with previous studies

The result of this study is similar with that of two previous meta-analyses. The first meta-analysis by Stopsack *et al* [24] found metformin use was associated with improved OS and RFS for patients with PCa by meta-analysing 9 studies. By pooling 8 studies, Hwang *et al* [25] found that PCa patients who used metformin had RFS benefits compared with those who did not use metformin. However, due to small number of included studies and limited sample size, no statistical significance was found for other outcomes such as CSS. For the present meta-analysis, we have tried to explore the potential between-study heterogeneity by conducting subgroup analyses in terms of OS subset. Though no significant decrease in heterogeneity of the subgroups, we still could not exclude the potential heterogeneity from these origins. Moreover, the trim-and-fill method further confirmed the robustness of results for OS and CSS. However, it do add the implications that metformin could influence survival in specific individuals with PCa, not in others. We found that metformin use might have overall survival effects in selected patients and well-designed studies, such as in patients involving only diabetics and metformin use calculated as ever versus never use, etc. This really gives implications in future design of clinical interventional study.

### Potential mechanisms

Several potential mechanisms for the anti-neoplastic action of metformin have been noted. Metformin, as an activator of AMP-activated protein kinase (AMPK), may play an important role in cancer metabolism. AMPK pathway is reported to inhibit mTOR signaling and result in fatty acid synthesis, inhibition of protein synthesis, and cell proliferation [26]. It has been reported that fatty acid synthase is overexpressed in PCa, breast cancer and pancreatic cancer, which is necessary for de novo fatty acid biosynthesis and malignant phenotype. AMPK activation can reduce the expression of fatty acid synthase and acetyl-CoA carboxylase, which diminishes the metabolization and growth of PCa cells [27]. Zadra *et al* [28] also suggested that suppression of *de novo* lipogenesis affected AMPK-mediated inhibition of PCa growth. In addition, metformin plays a role in cyclin-dependent kinase (CDK) induction of autophagy, cell cycle arrest, and apoptosis. Metformin can reduce the activity of cyclin D1, leading to the inhibition of PCa cell lines [29]. It has been vertified that the cyclin D1 pathway can serve as a regulator of androgen-dependent transcription and cell cycle progression in PCa cells [30].

### Strengths and limitations of the study

There were several limitations in our study. First, the statistical analysis of publication bias was insufficiently powered due to the small number of included studies for OS (*n* = 8) and CSS (*n* = 6) subsets, although the results were adjusted by the trim-and-fill model. Secondly, the sensitivity analyses could not be carried out related to the tumor site, disease stage and follow-up period because of unavailability of these data from the included studies, and these factors can also affect the prognosis of PCa patients. Thirdly, the accuracy and precision of the summary estimates could be influenced by the different survival analysis approaches. Although most of the studies used multivariate Cox proportional hazards model, other studies did not report the statistical models [17, 20], while another study did not utilize multivariate analysis [11]. In addition, adjustment variables between the included studies are not completely consistent. Fourthly, we were not able to contact the authors or sponsors of some studies to retrieve the data which were excluded from our analyses [12, 20]. This might lead to publication bias for pooled estimates.

Several important strengths of our study are presented as follows. Firstly, we performed a comprehensive search of the relevant studies in several main databases without language, publication date or publication type (both full text and abstract) limits, enabling us to select the maximal number of suitable studies for analysis. Secondly, the large sample size including over 100,000 individuals enabled us to quantitatively assess the association between metformin use and PCa prognosis, making it the most powerful and comprehensive synthesis of the evidence on this issue to date. Thirdly, we performed appropriate subgroup analyses for some key study characteristics, such as the study design, study setting, and Newcastle-Ottawa scale (NOS) scale for study quality. Fourthly, we selected and cross-checked the identified studies, developed the data abstraction forms, abstracted the data and assessed the study quality at least by two independent authors to avoid subjectivity to the greatest extent, making the process of the systematic review more objectively.

In summary, our current systematic review and meta-analysis found that metformin was beneficial for survival in patients with PCa, although the true association still need further confirmation based on the existing evidence. Nevertheless, this report indeed provides a direction for clinicians in the treatment of PCa. In future, larger prospective cohort studies, or even randomized controlled trials with longer follow-up period are needed to confirm the associations between metformin intake and PCa survival.

## MATERIALS AND METHODS

### Literature search

A search strategy in line with the preferred reporting items for systematic reviews and meta-analysis (PRISMA) statement was developed [31]. We performed systematic literature searches of PubMed, Embase and Cochrane databases from inception through January 2017 which investigated associations between metformin use and PCa prognosis. Supplementary Tables 1–3 present the above three database search strategies by using the combinations of following terms: ‘metformin’, ‘biguanides’, ‘prostate’, ‘prostatic’, ‘cancer’, ‘carcinoma’, ‘mortality’, ‘prognosis’, ‘prognostic’ and ‘survival’. We also performed manual reference search of the reference lists from the initial identified relevant studies, reviews and meta-analysis. We restricted the publication language only to English language studies, given the fact that studies published in other languages were often not available for both authors and readers.

### Study selection

Two authors (Liu and Chu) independently assessed the searched all the citations through the primary literature search, then identified the final relevant studies for eligibility. Agreement was reached for the discrepancies through discussion or by a senior author (Hao or Xu) if necessary. Studies were considered eligible for inclusion if the following criteria were met: prospective or retrospective cohort studies reported prognostic effects in PCa patients comparing metormin users with non-users, and survival estimates HRs/ risk ratios (RRs) with 95% CIs could be abstracted or calculated using the method reported by Parmar [32]. We used the most detailed or recent information for publications with overlapped data.

### Data extraction

The characteristics of each study included were extracted including the first author, publication year, study region and design, study setting, hospital number involved, sample size, follow-up duration, survival endpoints, and HRs or RRs with corresponding 95% CIs and adjusted variables.  

### Quality assessment

Methodological quality assessment for each study included was performed by two authors (Liu and Chu) and was scored them using the NOS [33]. The two authors scored the study quality of reviewed studies independently, and reach a consensus value for each item.

### Statistical analysis

All analyses were performed by using STATA 12.0 (StataCorp LP, College Station, TX). Survival estimates (HRs/RRs with 95% CIs) with full adjustments were abstracted from the included studies and pooled using random-effects model [34]. An observed HR < 1 implied an improved survival for the group with metformin use. The HRs for the study endpoints of OS, CSS and RFS were pooled separately. Between-study heterogeneity was assessed using *I*^2^ statistic and the Cochrane Q statistic, defined as an *I*^2^-value > 50% and *p*-value < 0.10 indicating substantial heterogeneity, respectively [35].

To further explore the potential heterogeneity, we performed subgroup analyses by investigating potential influencial variables that could explain some of the heterogeneity. Subgroup differences were calculated using the methods described by Deeks *et al* [36].

Publication bias was assessed by visual inspection of a funnel plot symmetry and using methods reported by Egger *et al* and Begg *et al* [37, 38]. We also examined the potential effect of publication bias through Duval’s nonparametric trim-and-fill method [39] to adjust the pooled HR.

## SUPPLEMENTARY MATERIALS TABLES


