# Supplementary material for: The impact of metformin use on survival in prostate cancer: a systematic review and meta-analysis
Source: Oncotarget. 2017 Oct 31;8(59):100449–58. doi: 10.18632/oncotarget.22117 (PMC5725033; doi:10.18632/oncotarget.22117)
Supplement: Supplementary file 1 [file oncotarget-08-100449-s001.pdf]

# The impact of metformin use on survival in prostate cancer: a systematic review and meta-analysis

## SUPPLEMENTARY MATERIALS

### Search Strategy

Search included: Pubmed, Embase, the Cochrane Library Central Register of Controlled Trials and American Society of Clinical Oncology (ASCO): until January 14 2017

**Supplementary Table 1: Search strategy for Pubmed (Publication date to 2017/01/14)**

| |
|---|
| 1. "Prostatic Neoplasms"[Mesh] |
| 2. (prostate[Title/Abstract]) OR prostatic[Title/Abstract] |
| 3. (Cancer OR tumor OR tumour OR carcinoma OR neoplas* OR malignan* OR phyma)[Title/Abstract] |
| 4. 2 AND 3 |
| 5. 1 OR 4 |
| 6. "Metformin"[Mesh] OR "Biguanides"[Mesh] |
| 7. (Metformin OR Dimethylbiguanidine OR Dimethylguanylguanidine OR Glucophage OR biguanide) [Title/Abstract] |
| 8. 6 OR 7 |
| 9. "Mortality"[Mesh] |
| 10. "Survival"[Mesh] |
| 11. "Prognosis"[Mesh] |
| 12. ((((((prognos*[Title/Abstract]) OR survival[Title/Abstract]) OR recurren*[Title/Abstract]) OR mortality[Title/Abstract]) OR predict*[Title/Abstract]) OR outcome*[Title/Abstract]) OR death[Title/Abstract] |
| 13. 9 OR 10 OR 11 OR 12 |
| 14. 5 AND 8 AND 13 |

Results 111

**Supplementary Table 2: Search strategy for Embase (Publication date to 2017/01/14)**

| |
|---|
| 1. ‘’prostate cancer’/exp |
| 2. ((Prostate OR prostatic) AND (Cancer OR tumor OR tumour OR carcinoma OR neoplas* OR malignan* OR phyma)):ab,ti |
| 3. 1 OR 2 |
| 4. ‘metformin’/exp |
| 5. ‘biguanide derivative’/exp |
| 6. (Metformin OR Dimethylbiguanidine OR Dimethylguanylguanidine OR Glucophage OR biguanide):ab,ti |
| 7. 4 OR 5 OR 6 |
| 8. ‘mortality’/exp |
| 9. ‘survival’/exp |
| 10. ‘prognosis’/exp |
| 11. (prognos* OR survival OR recurren* OR mortality OR predict* OR outcome* OR death):ab,ti |
| 12. 8 OR 9 OR 10 OR 11 |
| 13. 3 AND 7 AND 12 |

Results 438

**Supplementary Table 3: Search strategy for the Cochrane Library Central Register of Controlled Trials (Publication date to 2017/01/14)**

| |
|---|
| 1. MeSH descriptor: [Prostatic Neoplasms] explode all trees |
| 2. Prostate or prostatic:ti,ab,kw (Word variations have been searched) |
| 3. Cancer or tumor or tumour or carcinoma or neoplas* or malignan* or phyma:ti,ab,kw (Word variations have been searched) |
| 4. 2 AND 3 |
| 5. 1 OR 4 |
| 6. MeSH descriptor: [Metformin] explode all trees |
| 7. MeSH descriptor: [Biguanides] explode all trees |
| 8. Metformin or Dimethylbiguanidine or Dimethylguanylguanidine or Glucophage or biguanide:ti,ab,kw (Word variations have been searched) |
| 9. 6 OR 7 OR 8 |
| 10. MeSH descriptor: [Mortality] explode all trees |
| 11. MeSH descriptor: [Survival] explode all trees |
| 12. MeSH descriptor: [Prognosis] explode all trees |
| 13. prognos* or survival or recurren* or mortality or predict* or outcome* or death:ti,ab,kw (Word variations have been searched) |
| 14. 10 OR 11 OR 12 OR 13 |
| 15. 5 AND 9 AND 14 |

Results 12

**Supplementary Table 4: Selection procedure of included and excluded studies**

| | Reference list no. |
|---|---|
| Studies included in the meta-analysis (*n* = 13) | 1–13 |
| Studies excluded from meta-analysis, and reasons are listed below (*n* = 94) | 14–108 |
| **Reason for exclusion** | |
| Publication with cases included in a selected study (*n* =3) | 14–16 |
| No prognostic outcomes recorded (*n* =63) | 17–79 |
| Letters, comments, reviews or meta-analyses (*n* =26) | 80–105 |
| No sufficient data for analysis (*n* =3) | 106–108 |

**Supplementary Table 5: Meta-analysis of metformin use and patient survival outcomes; analyses of the publication bias with different models for the overall survival, cancer-specific survival and recurrence-free survival**

| Variable | No of studies | HR (95% CI) | $I^2$, Phet |
|---|---|---|---|
| Overall survival | 8 | 0.79 (0.63 to 0.98) | 79.5, <0.001 |
| Cancer-specific survival | 6 | 0.76 (0.57 to 1.02) | 65.3, 0.013 |
| Recurrence-free survival | 5 | 0.74 (0.58 to 0.95) | 29.0, 0.228 |

| Publication bias | Begg's *P* value | Egger's *P* value | T&F(Fill) |
|---|---|---|---|
| Overall survival | 1.00 | 0.69 | 0.79 (0.63 to 0.98) (0) |
| Cancer-specific survival | 0.26 | 0.32 | 0.76 (0.57 to 1.02) (0) |
| Recurrence-free survival | 0.22 | 0.24 | 0.74 (0.58 to 0.95) (0) |

Abbreviations: CI, confidence interval; Fill, number of studies added by trim and fill method; het, heterogeneity; HR, hazard ratio; T&F, result of trimmed and filled analysis, using assumption of random effects.

## REFERENCES

1. Mayer MJ, Klotz LH, Venkateswaran V. The Effect of Metformin Use during Docetaxel Chemotherapy on Prostate Cancer Specific and Overall Survival of Diabetic Patients with Castration Resistant Prostate Cancer. J Urol. 2017; 197:1068–75.
2. Chong RW, Vasudevan V, Zuber J, Solomon SS. Metformin Has a Positive Therapeutic Effect on Prostate Cancer in Patients With Type 2 Diabetes Mellitus. Am J Med Sci. 2016; 351:416–9.
3. Reznicek D, Klyushnenkova E, Alexander R. Metformin use predicts an overall survival advantage in diabetic veterans with prostate cancer. J Urol. 2015; 193:e146–e7.
4. Randazzo M, Beatrice J, Huber A, Grobholz R, Manka L, Wyler SF, Chun FF, Recker F, Kwiatkowski M. Influence of metformin use on PSA values, free-to-total PSA, prostate cancer incidence and grade and overall survival in a prospective screening trial (ERSPC Aarau). World J Urol. 2015; 33:1189–96.
5. Lu-Yao GL, Lin Y, Moore D, Graff J, Stroup A, McGuigan K, Crystal S, Amin S, Demissie K, DiPaola RS. Combination statin/metformin and prostate cancer specific mortality: A population-based study. J Clin Oncol. 2015; 33.
6. Lee H, Kuk H, Byun SS, Lee SE, Hong SK. Preoperative glycemic control status as a significant predictor of biochemical recurrence in prostate cancer patients after radical prostatectomy. PloS One. 2015; 10:e0124761.
7. Kaushik D, Karnes RJ, Eisenberg MS, Rangel LJ, Carlson RE, Bergstralh EJ. Effect of metformin on prostate cancer outcomes after radical prostatectomy. Urol Oncol. 2014; 32:43.e1–7.
8. Bensimon L, Yin H, Suissa S, Pollak MN, Azoulay L. The use of metformin in patients with prostate cancer and the risk of death. Cancer Epidemiol Biomarkers Prev. 2014; 23:2111–8.
9. Spratt DE, Zhang C, Zumsteg ZS, Pei X, Zhang Z, Zelefsky MJ. Metformin and prostate cancer: reduced development of castration-resistant disease and prostate cancer mortality. Eur Urol. 2013; 63:709–16.
10. Margel D, Urbach DR, Lipscombe LL, Bell CM, Kulkarni G, Austin PC, Fleshner N. Metformin use and all-cause and prostate cancer-specific mortality among men with diabetes. J Clin Oncol. 2013; 31:3069–75.
11. Spratt D, Zhang C, Zumsteg ZS, Pei X, Zhang Z, Yamada J, Kollmeier M, Cox B, Zelefsky MJ. Metformin improves prostate cancer-specific survival and inhibits the development of castrate resistant metastasis. Int J Radiat Oncol Biol Phys. 2012; 84:S93.
12. He XX, Tu SM, Lee MH, Yeung SC. Thiazolidinediones and metformin associated with improved survival of diabetic prostate cancer patients. Ann Oncol. 2011; 22:2640–5.
13. Patel T, Hruby G, Abate-Shen C, Badani K, McKiernan J. The impact of diabetes and metformin use in clinically localized prostate cancer treated by radical prostatectomy. J Urol. 2010; 183:e102.
14. Bensimon L, Yin H, Suissa S, Pollak MN, Azoulay L. Metformin use in prostate cancer and the risk of death and metastasis in patients with type 2 diabetes. Pharmacoepidemiol Drug Saf. 2013; 22:110.
15. Mayer M, Klotz L, Venkateswaran V. Diabetic metastatic castration-resistant prostate cancer patients administered metformin during docetaxel chemotherapy have improved prostate cancer-specific and overall survival. Cancer Res. 2016; 76.
16. Chong RW, Vasudevan V, Zuber J, Solomon S. Metformin has a positive therapeutic effect on prostate cancer in DM2 patients. J Investig Med. 2015; 63:357.
17. Haggstrom C, Van Hemelrijck M, Zethelius B, Robinson D, Grundmark B, Holmberg L, Gudbjornsdottir S, Garmo H, Stattin P. Prospective study of Type 2 diabetes mellitus, anti-diabetic drugs and risk of prostate cancer. Int J Cancer. 2017; 140:611–7.
18. Xie Y, Wang L, Hussain A. Metformin enhances the anti-prostate cancer activity of abiraterone and enzalutamide. Cancer Res. 2016; 76.
19. Tran LNK, Kichenadasse G, Morel KL, Ormsby RJ, Butler LM, Centenera MM, Sykes PJ. Combination of metformin and valproic acid in personalized prostate cancer treatment: The role of p53 and androgen receptor signaling. Cancer Res. 2016; 76.
20. Roberts MJ, Yaxley JW, Coughlin GD, Gianduzzo TRJ, Esler RC, Dunglison NT, Chambers SK, Medcraft RJ, Chow CWK, Schirra HJ, Richards RS, Kienzle N, Lu M, et al. Can atorvastatin with metformin change the natural history of prostate cancer as characterized by molecular, metabolomic, imaging and pathological variables? A randomized controlled trial protocol. Contemp Clin Trials. 2016; 50:16–20.
21. McCormick JR, Blute ML Jr, Yang B, Damaschke N, Jarrard DF. Synthetic lethal metabolic targeting of cellular senescence in prostate cancer with the repurposed drug metformin. J Urol. 2016; 195:e673–e4.
22. Marcinko K, Villani L, Houde V, Muti P, Steinberg G, Tsakiridis T. Combined salicylate (SAL)-metformin (MET) treatment induces increased tumour suppression and radiosensitization in preclinical models of prostate cancer (PRCA). Radiother Oncol. 2016; 120:S35.
23. Isebaert S, Gonnissen A, McKee C, Muschel R, Haustermans K. Combining Hedgehog inhibition with metformin to induce radiosensitization in prostate cancer cells. Radiother Oncol. 2016; 119:S960.
24. Hill J, Paulden M, McCabe C, Venner P, Danielson BL, North SA, Usmani N. Metformin (Met): A cost-effective adjunct therapy with enzalutamide (Enza) for metastatic castrate-resistant prostate cancer (mCRPC)? J Clin Oncol. 2016; 34.
25. Whitburn J, Aylward J, Webb S, Rao S, Hamdy F, Edwards C. The effect of metformin on osteomimicry and cancerinduced bone disease in the prostate cancer-bone microenvironment *in vitro* and *in vivo*. J Urol. 2015; 193:e822.

26. Tsutsumi Y, Nomiyama T, Kawanami T, Hamaguchi Y, Tanaka T, Yanase T. Combining exendin-4 treatment with metformin attenuates prostate cancer growth. Diabetes. 2015; 64:A525.

27. Shao C, Ahmad N, Hodges K, Kuang S, Ratliff T, Liu X. Inhibition of polo-like kinase 1 (Plk1) enhances the antineoplastic activity of metformin in prostate cancer. J Biol Chem. 2015; 290:2024–33.

28. Rhee H, Vela I, Gunter J, Jovanovic L, Hollier B, Williams E, Wood S, Ho K, Nelson C. Clinical application of biomarkers in prostate cancer in men with metabolic syndrome project: Clinical evaluation of prognostic and metabolic benefits of Metformin in prostate cancer. BJU Int. 2015; 115:36.

29. Randazzo M, Hermanns T, Josef B, Poyet C, Huber A, Grobholz R, Manka L, Wyler S, Recker F, Kwiatkowski M. Association of metformin use on prostate cancer incidence in a prospective screening trial. Eur Urol Suppl. 2015; 14:e15.

30. Randazzo M, Hermanns T, Poyet C, Beatrice J, Huber A, Grobholz R, Manka L, Wyler S, Recker F, Kwiatkowski M. Association of metformin use with prostate cancer incidence in a prospective screening trial (ERSPC Aarau). J Urol. 2015; 193:e1078.

31. Murtola TJ, Wahlfors T, Haring A, Taari K, Stenman UH, Tammela TL, Schleutker J, Auvinen A, and PRACTICAL Consortium. Polymorphisms of Genes Involved in Glucose and Energy Metabolic Pathways and Prostate Cancer: Interplay with Metformin. Eur Urol. 2015; 68:1089–97.

32. Murtola TJ, Wahlfors T, Haring A, Taari K, Stenman U, Tammela TL, Schleutker J, Auvinen A. 13 Polymorphisms in genes of the glucose- and energy-metabolism pathways and prostate cancer: interplay with metformin. Eur Urol Suppl. 2015; 14:e13.

33. Murtola T, Wahlfors T, Haring A, Taari K, Stenman UH, Tammela T, Schleutker J, Auvinen A. Polymorphisms in genes of the glucose-and energymetabolism pathways and prostate cancer: Interplay with metformin. J Urol. 2015; 193:e149.

34. Merrick GS, Bennett A, Couture T, Butler WM, Galbreath RW, Adamovich E. Metformin Does Not Predict for Prostate Cancer Diagnosis, Grade, or Volume of Disease After Transperineal Template-guided Mapping Biopsy. Am J Clin Oncol. 2017; 40:353–57.

35. Joerger M, van Schaik RH, Becker ML, Hayoz S, Pollak M, Cathomas R, Winterhalder R, Gillessen S, Rothermundt C. Multidrug and toxin extrusion 1 and human organic cation transporter 1 polymorphisms in patients with castration-resistant prostate cancer receiving metformin (SAKK 08/09). Prostate Cancer Prostatic Dis. 2015; 18:167–72.

36. Gunter JH, Kate Philp L, Sarkar P, Hollier B, Sadowski MC, Lehman M, Nelson C. Insulin increases apoptotic resistance and metabolic capacity in prostate cancer cells, that is inhibited by metformin. Endocr Rev. 2015; 36.

37. Feng T, Sun X, Howard LE, Vidal AC, Gaines AR, Moreira DM, Castro-Santamaria R, Andriole GL, Freedland SJ. Metformin use and risk of prostate cancer: results from the REDUCE study. Cancer Prev Res (Phila). 2015; 8:1055–60.

38. Dirat B, Ader I, Golzio M, Massa F, Mettouchi A, Laurent K, Larbret F, Malavaud B, Cormont M, Lemichez E, Cuvillier O, Tanti JF, Bost F. Inhibition of the GTPase Rac1 mediates the antimigratory effects of metformin in prostate cancer cells. Mol Cancer Ther. 2015; 14:586–96.

39. Bilen MA, Lin SH, Tang DG, Parikh K, Lee MH, Yeung SC, Tu SM. Maintenance Therapy Containing Metformin and/or Zyflamend for Advanced Prostate Cancer: A Case Series. Case Rep Oncol Med. 2015; 2015:471861.

40. Babcook MA, Sramkoski RM, Fujioka H, Daneshgari F, Almasan A, Shukla S, Gupta S. Combination simvastatin and metformin induces G1-phase cell cycle arrest and Ripk1-and Ripk3-dependent necroptosis in C4-2B osseous metastatic castration-resistant prostate cancer cells. Cancer Res. 2015; 75.

41. Zhang J, Shen C, Wang L, Ma Q, Xia P, Qi M, Yang M, Han B. Metformin inhibits epithelial-mesenchymal transition in prostate cancer cells: involvement of the tumor suppressor miR30a and its target gene SOX4. Biochem Biophys Res Commun. 2014; 452:746–52.

42. Zhang T, Zhang L, Zhang T, Fan J, Wu K, Guan Z, Wang X, Li L, Hsieh JT, He D, Guo P. Metformin sensitizes prostate cancer cells to radiation through EGFR/p-DNA-PKCS *in vitro* and *in vivo*. Radiat Res. 2014; 181:641–9.

43. Xu H, Hu MB, Bai PD, Zhu WH, Ding Q, Jiang HW. Will metformin postpone high-fat diet promotion of TRAMP mouse prostate cancer development and progression? Int Urol Nephrol. 2014; 46:2327–34.

44. Tseng CH. Metformin significantly reduces incident prostate cancer risk in Taiwanese men with type 2 diabetes mellitus. Eur J Cancer. 2014; 50:2831–7.

45. Rothermundt C, Hayoz S, Templeton AJ, Winterhalder R, Strebel RT, Bartschi D, Pollak M, Lui L, Endt K, Schiess R, Ruschoff JH, Cathomas R, Gillessen S. Metformin in chemotherapy-naive castration-resistant prostate cancer: a multicenter phase 2 trial (SAKK 08/09). Eur Urol. 2014; 66:468–74.

46. Preston MA, Riis AH, Ehrenstein V, Breau RH, Batista JL, Olumi AF, Mucci LA, Adami HO, Sorensen HT. Metformin use and prostate cancer risk. Eur Urol. 2014; 66:1012–20.

47. Joshua AM, Zannella VE, Downes MR, Bowes B, Hersey K, Koritzinsky M, Schwab M, Hofmann U, Evans A, van der Kwast T, Trachtenberg J, Finelli A, Fleshner N, et al. A pilot 'window of opportunity' neoadjuvant study of metformin in localised prostate cancer. Prostate Cancer Prostatic Dis. 2014; 17:252–8.

48. Demir U, Koehler A, Schneider R, Schweiger S, Klocker H. Metformin anti-tumor effect via disruption of the MID1 translational regulator complex and AR downregulation in prostate cancer cells. BMC Cancer. 2014; 14:52.

49. Babcook MA, Sramkoski RM, Oak CZ, Gupta S. Combination simvastatin and metformin induces cell death by autophagy and secondary necrosis in osseous metastatic castration-resistant prostate cancer cells. Cancer Res. 2014; 74.

50. Babcook MA, Sramkoski RM, Fujioka H, Daneshgari F, Almasan A. Combination simvastatin and metformin induces

G1-phase cell cycle arrest and Ripk1- and Ripk3-dependent necrosis in C4-2B osseous metastatic castration-resistant prostate cancer cells. 2014; 5:e1536.

51. Babcook MA, Shukla S, Fu P, Vazquez EJ, Puchowicz MA, Molter JP, Oak CZ, MacLennan GT, Flask CA, Lindner DJ, Parker Y, Daneshgari F, Gupta S. Synergistic simvastatin and metformin combination chemotherapy for osseous metastatic castration-resistant prostate cancer. Mol Cancer Ther. 2014; 13:2288–302.

52. Margel D, Urbach D, Lipscombe LL, Bell CM, Kulkarni G, Austin PC, Fleshner N. Association between metformin use and risk of prostate cancer and its grade. J Natl Cancer Inst. 2013; 105:1123–31.

53. Gunter J, Lubik A, Vasireddy R, Stylianou N, Hendy S, Hollier B, Gleave M, Pollak M, Herington A, Nelson C. Metformin modulates key insulin-induced survival pathways in prostate cancer cells. BJU Int. 2013; 112:40.

54. Gaisser A. Oncology and care in the specialty and public media-Mammography in breast implants, ginseng in cancer-related fatigue, colorectal cancer screening with capsule endoscopy, sipuleucel-T in prostate cancer, metformin and prognosis of prostate cancer. Onkologe. 2013; 1–3.

55. Fendt SM, Bell EL, Keibler MA, Davidson SM, Wirth GJ, Fiske B, Mayers JR, Schwab M, Bellinger G, Csibi A, Patnaik A, Blouin MJ, Cantley LC, et al. Metformin decreases glucose oxidation and increases the dependency of prostate cancer cells on reductive glutamine metabolism. Cancer Res. 2013; 73:4429–38.

56. Dal Pra A, Zannella V, Glicksman R, Sykes J, Muaddi H, Joshua AM, Wouters BG, Milosevic M, Koritzinsky M, Bristow RG. Metformin and prostate cancer radiation therapy: Improved outcomes due to enhanced tumor oxygenation. Int J Radiat Oncol Biol Phys. 2013; 87:S170.

57. Babcook MA, Sramkoski RM, Vazquez EJ, Puchowicz MA, Shukla S, Gupta S. Synergistic simvastatin and metformin chemotherapy for metastatic castration-resistant prostate cancer. Cancer Res. 2013; 73.

58. Akinyeke T, Matsumura S, Wang X, Wu Y, Schalfer ED, Saxena A, Yan W, Logan SK, Li X. Metformin targets c-MYC oncogene to prevent prostate cancer. Carcinogenesis. 2013; 34:2823–32.

59. Rothermundt CA, Cathomas R, Templeton A, Winterhalder RC, Strebel R, Baertschi D, Pollak M, Lui L, Crowe S, Gillessen S. Metformin in chemotherapy-naïve castration resistant prostate cancer (CRPC): A multicenter phase ii trial (SAKK 08/09). Ann Oncol. 2012; 23:ix302.

60. Nobes JP, Langley SE, Klopper T, Russell-Jones D, Laing RW. A prospective, randomized pilot study evaluating the effects of metformin and lifestyle intervention on patients with prostate cancer receiving androgen deprivation therapy. BJU Int. 2012; 109:1495–502.

61. Monteagudo S, Perez-Martinez FC, Perez-Carrion MD, Guerra J, Merino S, Sanchez-Verdu MP, Cena V. Inhibition of p42 MAPK using a nonviral vector-delivered siRNA potentiates the anti-tumor effect of metformin in prostate cancer cells. Nanomedicine (Lond). 2012; 7:493–506.

62. Gupta S, Singh A, Jagar P, Giese G, Patil S, Lad TE. Effect of prior metformin intake on first diagnosis of prostate cancer in diabetic men. Eur Urol Suppl. 2012; 11:206–7.

63. Colquhoun AJ, Venier NA, Vandersluis AD, Besla R, Sugar LM, Kiss A, Fleshner NE, Pollak M, Klotz LH, Venkateswaran V. Metformin enhances the antiproliferative and apoptotic effect of bicalutamide in prostate cancer. Prostate Cancer Prostatic Dis. 2012; 15:346–52.

64. Carney BK, Cassimeris L. Docetaxel and metformin act synergistically to inhibit growth of prostate cancer cell lines. Mol Biol Cell. 2012; 23.

65. Biernacka K, Perks C, Gillatt D, Persad R, Uzoh C, Holly J. Epigenetic reprogramming of IGFBP-2 in prostate cancer mediates hyperglycaemia-induced chemoresistance and the effect of metformin. Growth Horm IGF Res. 2012; 22:S74.

66. Akinyeke T, Wang X, Matsumura S, Shinglot H, Bhatt R, Saxena A, Yan W, Li X. Metformin targets tumor cells and the microenvironment to prevent prostate cancer initiation and growth in bone. J Bone Miner Res. 2012; 27.

67. Klotz L, Venier N, Vandersluis A, Besla R, Fleshner N, Pollak MN, Venkateswaran V, Colquhoun AJ. Utilizing metformin to enhance the efficacy of androgen-deprivation therapy in the treatment of prostate cancer. J Clin Oncol. 2011; 29.

68. Colquhoun A, Venier N, Vandersluis A, Fleshner N, Pollak M, Klotz L, Venkateswaran V. Utilizing metformin as a radiosensitizing agent in the treatment of prostate cancer. J Urol. 2011; 185:e296.

69. Colquhoun A, Venier N, Vandersluis A, Fleshner N, Pollak M, Klotz L, Venkateswaran V. Utilizing metformin to enhance the efficacy of androgen deprivation therapy in the treatment of prostate cancer. J Urol. 2011; 185:e293.

70. Colquhoun AJ, Venier NA, Fleshner NE, Pollak M, Klotz LH, Venkateswaran V. Utilizing metformin to enhance the efficacy of androgen deprivation therapy in the treatment of prostate cancer. Cancer Res. 2010; 70.

71. Colquhoun AJ, Venier NA, Vandersluis AD, Besla R, Fleshner NE, Pollak M, Klotz LH, Venkateswaran V. Utilising metformin as a radiosensitising agent in the treatment of prostate cancer. Eur Urol Suppl. 2011; 10:293.

72. Colquhoun AJ, Venier NA, Vandersluis AD, Besla R, Fleshner NE, Pollak M, Klotz LH, Venkateswaran V. Utilising metformin to enhance the efficacy of androgen deprivation therapy in the treatment of prostate cancer. Eur Urol Suppl. 2011; 10:293.

73. Colquhoun AJ, Venier NA, Fleshner NE, Pollak M, Klotz LH, Venkateswaran V. Utilising metformin to enhance the efficacy of androgen deprivation therapy in the treatment of prostate cancer. Eur Urol Suppl. 2010; 9:219.

74. Biernacka KM, Holly JMP, Uzoh C, Gillatt D, Persad R, Perks CM. Metformin counter-acts hyperglycaemiainduced chemoresistance of prostate cancer cells. Eur Urol Suppl. 2011; 10:293–4.

75. Azoulay L, Dell'Aniello S, Gagnon B, Pollak M, Suissa S. Metformin and the incidence of prostate cancer in patients with

type 2 diabetes. Cancer Epidemiol Biomarkers Prev. 2011; 20:337–44.

76. Biernacka K, Holly JMP, Uzoh C, Gillatt D, Persad R, Perks CM. Effects of metformin on hyperglycaemia-induced chemoresistance of DU145 and LNCaP prostate cancer cells. Growth Horm IGF Res. 2010; 20:S34.
77. Ben Sahra I, Tanti JF, Bost F. The combination of metformin and 2-deoxyglucose inhibits autophagy and induces AMPK-dependent apoptosis in prostate cancer cells. Autophagy. 2010; 6:670–1.
78. Ben Sahra I, Laurent K, Giuliano S, Larbret F, Ponzio G, Gounon P, Le Marchand-Brustel Y, Giorgetti-Peraldi S, Cormont M, Bertolotto C, Deckert M, Auberger P, Tanti JF, et al. Targeting cancer cell metabolism: the combination of metformin and 2-deoxyglucose induces p53-dependent apoptosis in prostate cancer cells. Cancer Res. 2010; 70:2465–75.
79. Nobes JP, Langley SE, Laing RW. Use of metformin and lifestyle intervention to prevent ADT-related metabolic syndrome in prostate cancer. J Clin Oncol. 2009; 27:5159.
80. Hankinson SJ, Fam M, Patel NN. A review for clinicians: Prostate cancer and the antineoplastic properties of metformin. Urol Oncol. 2017; 35:21–9.
81. Stopsack KH, Ziehr DR, Rider JR, Giovannucci EL. Metformin and prostate cancer mortality: a meta-analysis. Cancer Causes Control. 2016; 27:105–13.
82. Karabulut-Bulan Ö. A new insight into metformin action: Diabetes, prostate cancer, and ion channels. Marmara Pharm J. 2016; 20:216–23.
83. Gillessen S, Gilson C, James N, Adler A, Sydes MR, Clarke N, and STAMPEDE Trial Management Group. Repurposing Metformin as Therapy for Prostate Cancer within the STAMPEDE Trial Platform. Eur Urol. 2016; 70:906–8.
84. Raval AD, Thakker D, Vyas A, Salkini M, Madhavan S, Sambamoorthi U. Impact of metformin on clinical outcomes among men with prostate cancer: a systematic review and meta-analysis. Prostate Cancer Prostatic Dis. 2015; 18:110–21.
85. Moyad MA, Vogelzang NJ. Heart healthy equals prostate healthy and statins, aspirin, and/or metformin (S.A.M.) are the ideal recommendations for prostate cancer prevention. Asian J Androl. 2015; 17:783–91.
86. Mayer MJ, Klotz LH, Venkateswaran V. Metformin and prostate cancer stem cells: a novel therapeutic target. Prostate Cancer Prostatic Dis. 2015; 18:303–9.
87. Hwang IC, Park SM, Shin D, Ahn HY, Rieken M, Shariat SF. Metformin association with lower prostate cancer recurrence in type 2 diabetes: a systematic review and meta-analysis. Asian Pac J Cancer Prev. 2015; 16:595–600.
88. Deng D, Yang Y, Tang X, Skrip L, Qiu J, Wang Y, Zhang F. Association between metformin therapy and incidence, recurrence and mortality of prostate cancer: evidence from a meta-analysis. Diabetes Metab Res Rev. 2015; 31:595–602.
89. Zanders MM, Vissers PA, van de Poll-Franse LV. Association between metformin use and mortality in patients with prostate cancer: explained by confounding by indication? J Clin Oncol. 2014; 32:701.
90. Yu H, Yin L, Jiang X, Sun X, Wu J, Tian H, Gao X, He X. Effect of metformin on cancer risk and treatment outcome of prostate cancer: a meta-analysis of epidemiological observational studies. PloS One. 2014; 9:e116327.
91. Taneja SS. Re: metformin use and all-cause and prostate cancer-specific mortality among men with diabetes. J Urol. 2014; 191:1783.
92. Taneja SS. Re: Metformin use and all-cause and prostate cancer-specific mortality among men with diabetes: Editorial comment. J Urol. 2014; 191:1783.
93. Hamilton RJ. Metformin for castrate-resistant prostate cancer: Learning more about an old dog’s new tricks. Eur Urol. 2014; 66:475–8.
94. Clyne M. Prostate cancer: metformin--the new wonder drug? Nat Rev Urol. 2014; 11:366.
95. Clyne M. Prostate cancer: Should we give metformin to all men with CRPC? Nat Rev Urol. 2014; 11:63.
96. Clyne M. Prostate cancer: Metformin - The new wonder drug? Nat Rev Urol. 2014; 11:366.
97. Babcook M, Shukla S, Sramkoski M, Fu P, Vazquez E, Puchowicz M, Oak C, Molter J, MacLennan G, Flask C, Lindner D, Parker Y, Gupta S. Simvastatin and metformin: A deadly combination for metastatic castration-resistant prostate cancer. J Urol. 2014; 191:e766.
98. Spratt DE, Zhang Z, Zelefsky MJ. Reply to Leah Bensimon, Samy Suissa, and Laurent Azoulay's letter to the editor re: Daniel E. Spratt, Chi Zhang, Zachary S. Zumsteg, Xin Pei, Zhigang Zhang, Michael J. Zelefsky. metformin and prostate cancer: reduced development of castration-resistant disease and prostate cancer mortality. Eur Urol. 2013; 63:709–16. Eur Urol. 2013; 64:e29–30.
99. Penney KL, Stampfer MJ. Time is ripe for a randomized trial of metformin in clinically localized prostate cancer. J Clin Oncol. 2013; 31:3054–5.
100. Murtola TJ, Wahlfors T, Tammela TLJ, Määttänen L, Schleutker J. SNPs in genes of the glucose-metabolism pathway and prostate cancer: Interplay with metformin. Cancer Prev Res. 2013; 6.
101. Downes MR, Sweet J, Zannella V, Bowes B, Koritzinsky M, Evans AJ, Trachtenberg J, Jewett M, Finelli A, Fleshner N, Pollak M, Joshua AM. The effects of metformin on the mTORC pathway in prostate cancer. Lab Invest. 2013; 93:207A.
102. Bensimon L, Suissa S, Azoulay L. Re: Daniel E. Spratt, Chi Zhang, Zachary S. Zumsteg, Xin Pei, Zhigang Zhang, Michael J. Zelefsky. Metformin and prostate cancer: reduced development of castration-resistant disease and prostate cancer mortality. Eur Urol. 2013; 63:709–16. Eur Urol. 2013; 64:e28.
103. Wirth GJ, Fendt SM, Bell EL, Keibler MA, Stephanopoulos G, Olumi AF. Metformin shifts prostate cancer metabolism towards a less malignant phenotype. Cancer Res. 2012; 72.
104. Moyad MA. Re: A prospective, randomized pilot study evaluating the effects of metformin and lifestyle intervention on patients with

prostate cancer receiving androgen deprivation therapy. Eur Urol. 2012; 61:623–24.

105. Clements A, Gao B, Yeap SH, Wong MK, Ali SS, Gurney H. Metformin in prostate cancer: two for the price of one. Ann Oncol. 2011; 22:2556–60.

106. Rodrigues V, Dias E, Mota P, Cordeiro A, Botelho F. Use of acetylsalicylic acid, metformin and statins and prostate cancer: Impact on the pathological characteristics and risk of biochemical recurrence. Acta Urologica Portuguesa. 2015; 32:78–85.

107. Taira AV, Merrick GS, Galbreath RW, Morris M, Butler WM, Adamovich E. Metformin is not associated with improved biochemical free survival or cause-specific survival in men with prostate cancer treated with permanent interstitial brachytherapy. J Contemp Brachytherapy. 2014; 6:254–61.

108. Rieken M, Kluth LA, Xylinas E, Fajkovic H, Becker A, Karakiewicz PI, Herman M, Lotan Y, Seitz C, Schramek P, Remzi M, Loidl W, Pummer K, et al. Association of diabetes mellitus and metformin use with biochemical recurrence in patients treated with radical prostatectomy for prostate cancer. World J Urol. 2014; 32:999–1005.
